# Contemporary surgical strategies for pediatric laryngotracheal stenosis: a comprehensive review

**DOI:** 10.3389/fped.2025.1634634

**Published:** 2025-08-26

**Authors:** Hanne Oscé, Jeroen Meulemans, Greet Hens

**Affiliations:** Department of Otorhinolaryngology, Head and Neck Surgery, University Hospitals Leuven, Leuven, Belgium

**Keywords:** pediatric airway, laryngotracheal stenosis, laryngotracheal reconstruction, cricotracheal resection, airway surgery, subglottic stenosis

## Abstract

**Background:**

Pediatric laryngotracheal stenosis (LTS) presents a complex and heterogeneous clinical challenge, requiring individualized surgical approaches to restore airway patency and function. Depending on stenosis severity, anatomical site, and comorbidities, a range of surgical techniques—including laryngotracheal reconstruction (LTR), partial cricotracheal resection (PCTR), extended PCTR, and endoscopic posterior cricoid split with rib grafting (EPCS/RG)—may be employed.

**Methods:**

This narrative review synthesizes current surgical strategies for pediatric LTS based on current literature, highlighting their indications, operative considerations, and reported outcomes. Key factors affecting surgical success, such as patient selection and perioperative management, are discussed.

**Results:**

Single-stage LTR is favored in healthy children with moderate SGS, while PCTR offers superior outcomes in severe or recurrent cases. EPCS/RG represents a minimally invasive alternative for LTR in selected cases with posterior glottic stenosis. Decannulation rates generally exceed 85% in carefully selected patients, though voice and swallowing outcomes vary by technique.

**Conclusion:**

Optimal management of pediatric LTS requires a multidisciplinary, tailored approach. Continued focus on long-term functional outcomes, technical innovations, and multicenter collaboration will further improve patient care.

## Introduction

Pediatric laryngotracheal stenosis (LTS) is defined as a narrowing of the airway at the glottic, subglottic, or tracheal level, with the subglottis being the most frequently affected site. Among the various forms of subglottic stenosis (SGS), congenital SGS accounts for approximately 10% of cases and results from incomplete recanalization of the laryngeal lumen during the 10th week of gestation. Congenital cases may present as membranous or cartilaginous narrowing (1). In contrast, acquired SGS—more commonly encountered—is typically associated with iatrogenic injury following prolonged endotracheal intubation. Risk factors include the size and movement of the endotracheal tube, duration of intubation, traumatic intubation, concomitant infections, and conditions such as gastroesophageal reflux (1).

Clinical presentation varies depending on the severity of the stenosis. While severe cases may manifest at birth with stridor and respiratory distress, milder forms often present later in infancy or early childhood with nonspecific symptoms such as dyspnea, recurrent croup, feeding difficulties, or changes in voice. Management requires a patient-centered approach that considers not only the anatomical characteristics of the stenosis—its length, location, and severity—but also the child's age, comorbidities, and overall health status.

Mild stenoses are often managed endoscopically using carbon dioxide (CO₂) laser ablation, balloon dilations, topical mitomycin C, or intralesional steroid injections. However, the clinical benefit of mitomycin C remains uncertain, as there is limited scientific evidence to support its effectiveness in treating LTS. Furthermore, these interventions frequently require multiple sessions and may yield only temporary relief (2). Endoscopic techniques also serve a valuable role in addressing postoperative complications such as granulation tissue formation or restenosis.

In children with moderate to severe stenosis, or in those for whom endoscopic measures have failed or are unlikely to succeed, open surgical intervention becomes necessary. Open surgical techniques for pediatric laryngotracheal stenosis (LTS) can be broadly categorized into expansion procedures using grafts and segmental resections with end-to-end anastomosis. Among the available procedures, laryngotracheal reconstruction (LTR), partial cricotracheal resection (PCTR), extended PCTR, and endoscopic posterior cricoid split with rib grafting (EPCS/RG) remain the mainstays of surgical treatment. This review aims to provide an in-depth analysis of these contemporary surgical strategies, examining their indications, technical nuances, and outcomes, with a focus on improving long-term airway stability and functional recovery in pediatric patients.

## Preoperative work-up

A thorough preoperative evaluation is essential to optimize outcomes in pediatric airway surgery. Initial assessment should include a detailed medical history and physical examination, with attention to associated anomalies or comorbid conditions that may influence treatment planning. In complex cases, a multidisciplinary evaluation involving otolaryngology, pulmonology, cardiology, gastroenterology, anesthesiology, and intensive care is often warranted. Several factors critically influence surgical planning and outcomes, including vocal fold mobility, the presence of supraglottic or glottic scarring, cricoarytenoid joint fixation, coexisting airway lesions, and conditions such as obstructive sleep apnea (OSA), eosinophilic esophagitis, or gastroesophageal reflux disease (GERD) (3).

Awake flexible transnasal fiberoptic laryngoscopy is routinely performed to assess vocal fold mobility and screen for synchronous airway lesions such as laryngomalacia or tracheomalacia. Definitive assessment, however, is obtained through rigid direct laryngotracheobronchoscopy under general anesthesia with spontaneous ventilation. This remains the gold standard for evaluating the precise site, extent, thickness and severity of the stenosis. The examination typically includes systematic assessment from the supraglottis to the carina with 0° and 30° rigid endoscopes. In tracheostomized patients, particular attention is paid to the suprastomal area to identify collapse, granulation, or malacia. Stenosis severity is commonly classified using the modified Myer-Cotton grading system, which estimates the percentage of airway obstruction based on the largest endotracheal tube than can be passed through the stenotic segment (4). However, this system has limited predictive value for postoperative outcomes, particularly decannulation (5).

To address these limitations, the European Laryngological Society introduced a more comprehensive classification in 2015 (5). This staging system incorporates five steps assessing the number of anatomical subsites involved and considers comorbidities that may affect surgical outcomes. It has demonstrated higher prognostic accuracy concerning success after airway surgery, especially in patients undergoing PCTR (5).

When vocal fold immobility is present, suspension microlaryngoscopy can aid in distinguishing posterior glottic stenosis (PGS) from neurogenic causes of vocal fold paralysis. Laryngeal electromyography may offer additional diagnostic value. Airway cultures are routinely obtained preoperatively to guide perioperative antibiotic therapy. Imaging—such as computed tomography (CT) or magnetic resonance imaging (MRI)—is reserved for evaluating extralaryngeal extension, complex anatomy, or suspected vascular anomalies.

Pulmonary function testing and voice assessments (e.g., GRBAS scale, maximum phonation time) as well as swallowing evaluations—including aspiration risk—can be helpful but are often limited in younger children due to cooperation challenges. Therefore, these tests are not considered mandatory in routine preoperative evaluation.

Ultimately, surgical planning should be guided by multidisciplinary team discussion to determine the optimal approach based on airway pathology, comorbidities and institutional expertise.

## Surgical indications

### Laryngotracheal reconstruction

LTR remains a cornerstone in the treatment of mild-to-moderate SGS, particularly in patients with isolated, unilevel stenoses. Since its initial description by Cotton and Fearon in the 1970s, the technique has evolved to include various grafting options (6). Anterior grafts are typically used for mild SGS, posterior grafts for isolated posterior scarring, and combined anterior and posterior grafts for severe cases. In cases of extensive scarring (modified Cotton-Myer grade III and IV), outcomes are less favorable due to challenges such as impaired wound healing, granulation tissue formation due to limited residual mucosa, and subsequent risk of restenosis (7). LTR is also indicated in select cases of PGS, either in isolation or combined with SGS.

The choice between single-stage and double-stage LTR is determined by the patient's overall condition and surgical extent. Single-stage procedures, in which the tracheostomy is removed at the time of surgery, are favored in otherwise healthy children with limited disease. Double-stage LTR is preferred in children with comorbidities or when more extensive reconstruction is required.

### Partial cricotracheal resection and extended partial cricotracheal resection

PCTR is the preferred approach for advanced SGS (modified Cotton-Myer grade III or IV) or in cases with previous LTR failure (4). It involves circumferential resection of the stenotic segment and primary anastomosis of healthy trachea and cricoid. In case of minimal residual mucosa in the reconstructed subglottis, LTR may carry a higher risk of restenosis, requiring a second open procedure to achieve decannulation (8, 9). While PCTR can also be considered for select moderate stenoses, it is relatively contraindicated in low-grade SGS, in SGS with glottic involvement (within 3 mm of the vocal folds), or when tracheal mobilization is limited (4).

Single-stage PCTR is ideal for otherwise healthy children without glottic involvement, whereas a double-stage approach is chosen for more complex cases or those with significant comorbidities.

Extended PCTR combines PCTR with an additional posterior cricoid split and cartilage grafting and is reserved for severe SGS with glottic involvement, such as PGS, vocal fold synechia, webbing, or laryngeal atresia. This is generally a two-stage procedure.

### Endoscopic posterior cricoid split and costal cartilage graft insertion

EPCS/RG is an endoscopic technique used primarily for isolated PGS and, in some cases, SGS or bilateral vocal fold immobility (BVFI). The decision to use this technique depends on the severity and location of the stenosis, as well as whether the patient is tracheostomized.

Bogdasarian's classification helps guide treatment: grade I-II PGS may be treated endoscopically with CO_2_ laser and dilation; grade II with more fibrosis may require EPCS/RG; grade III-IV, characterized by arytenoid fixation, often necessitates tracheostomy and may be managed with EPCS/RG in tracheostomized patients or single-stage LTR in non-tracheostomized cases (10, 11).

## Surgical techniques

### Laryngotracheal reconstruction

LTR is performed to widen the glottic and subglottic airway by interposing cartilage grafts. Costal cartilage is the preferred graft material due to its strength, ease of carving, availability, and long-term stability. Thyroid and auricular cartilage may be used as alternative graft options. The operative approach follows the standardized technique described by Monnier and colleagues (12).

In tracheostomized patients, ventilation is initially managed through a flexo-metallic endotracheal tube passed via the tracheostoma. In non-tracheostomized children, anesthesia is induced with mask ventilation. During single-stage LTR, the tracheostoma is closed as part of the reconstruction; in double-stage procedures, the tracheostomy remains in place postoperatively.

A horizontal neck incision is made above the tracheostoma, followed by elevation of subplatysmal flaps and midline separation of the strap muscles. The thyroid isthmus is divided and mobilized laterally, while sparing the cricothyroid muscles. A midline vertical incision—the anterior laryngotracheofissure—is made through the lower portion of the thyroid lamina, cricoid cartilage, and upper two tracheal rings to expose the posterior cricoid plate, which is then divided in the midline, taking care to preserve the retrocricoid mucosa.

Costal cartilage is harvested through a separate 2–3 cm skin incision below the mammary line. The rib cartilage is dissected in a subperichondrial plane, preserving the outer perichondrium. Following hemostasis and saline irrigation, a Valsalva maneuver is used to exclude pneumothorax before closure.

**Figure 1:**
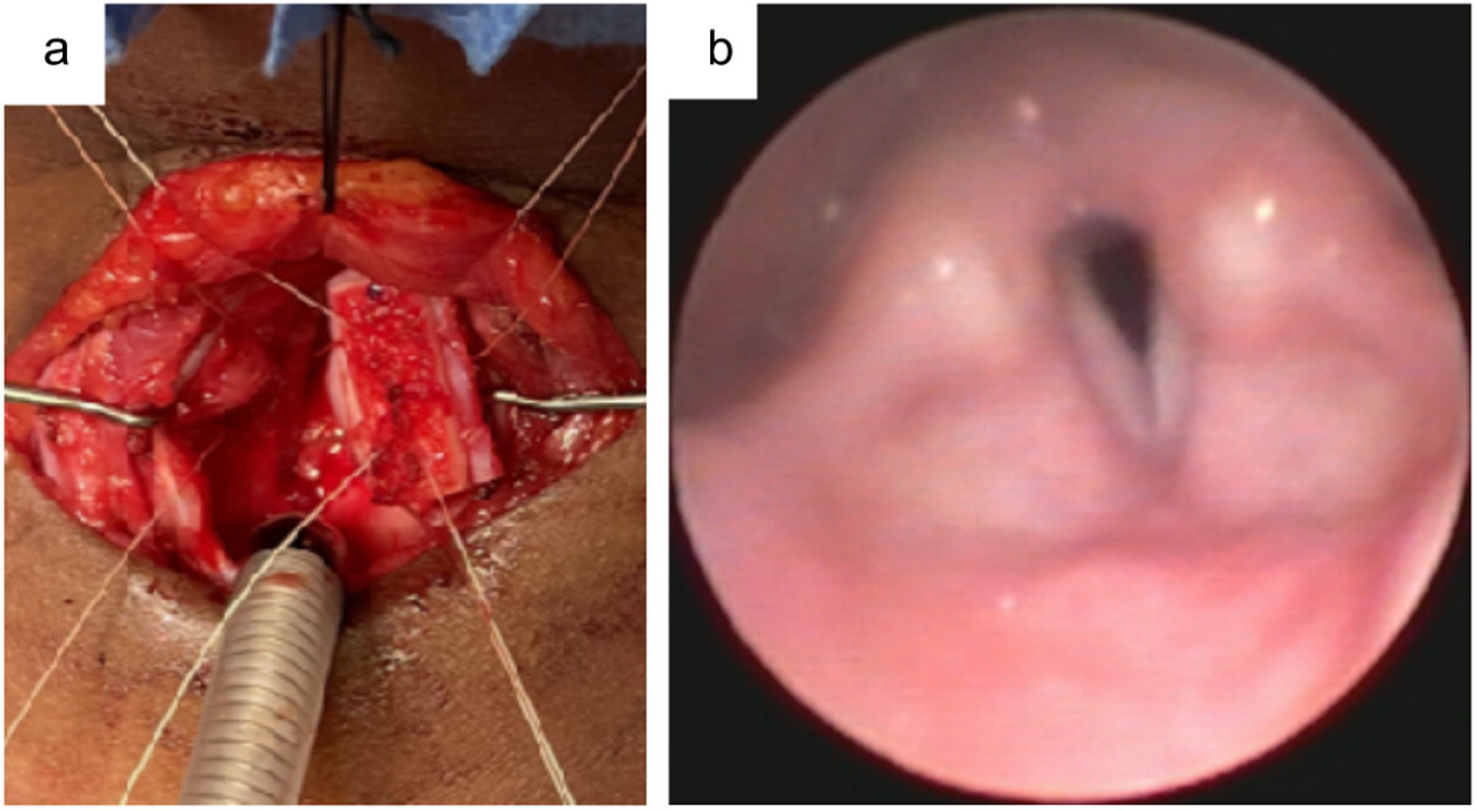
LTR in a 2.5-year-old female with PGS grade III. **(a)** A remodeled cartilage graft is positioned between the divided cricoid laminae using four guided 4.0 vicryl sutures. **(b)** A bronchoscopy performed six weeks postoperatively reveals a sufficient laryngeal lumen with some residual edema and limited vocal fold mobility, resulting in severe dysphonia, though the stridor had resolved.

The harvested graft is shaped based on the cranio-caudal height and thickness of the cricoid plate. According to Cotton's guideline, the graft width should approximate 1 mm per year of age, with a minimum of 4 mm (13). Overexpansion may result in anteroposterior collapse of the reconstructed airway. The graft is stabilized using 4.0 or 5.0 vicryl sutures passed through its perichondrial surface and anchored inside the airway. Fibrin sealant (e.g., Tisseel ®) is applied to the perichondrium to enhance stability (Figure 1).

Anterior cartilage grafting may also be performed to expand the airway and close the tracheostoma, if present. Meticulous fixation of anterior and posterior grafts is critical, especially in single-stage procedures where no stent is placed. In single-stage LTR, the patient is intubated nasotracheally for approximately seven days. In double-stage LTR, a stent (e.g., Montgomery T-tube or LT-Mold ®) is secured intralaryngeally with non-resorbable sutures before closure of the laryngotracheofissure.

The laryngotracheal incision is then closed with 4.0 vicryl sutures and application of fibrin sealant. A Penrose drain is positioned in the tracheal bed to prevent fluid accumulation. Closure involves reapproximation of the thyroid isthmus over the trachea, followed by midline suturing of the strap muscles. The skin is then closed in two layers.

### Partial cricotracheal resection and extended partial cricotracheal resection

PCTR involves circumferential resection of the stenotic subglottic segment and reanastomosis of healthy airway. This technique has become a mainstay for managing advanced SGS and is well described by Monnier and colleagues (12).

In tracheostomized children undergoing a double-stage procedure, anesthesia is administered via a flexo-metallic tube inserted through the tracheostoma. In non-tracheostomized patients, airway management often begins with mask ventilation, followed by intubation using the largest nasotracheal tube that can safely traverse the stenotic segment. In severe cases, definitive airway access may be achieved intraoperatively by advancing the nasotracheal tube into the distal airway after resection of the stenotic segment, thereby enabling safe ventilation during the anastomosis phase.

A horizontal neck incision is made at the level of the fourth tracheal ring. In tracheostomized children, a crescent of skin around the stoma is excised to allow mobilization. The subplatysmal skin flaps are then elevated, and the strap muscles are separated at the midline. The sternothyroid and thyrohyoid muscles are partially incised transversely at their thyroid cartilage insertion. The thyroid isthmus is then divided, allowing for stepwise dissection toward the cricoid and proximal trachea. Care is taken to preserve the recurrent laryngeal nerves (RLNs) by maintaining close dissection along the tracheal cartilaginous rings. Special attention is given to preserving the segmental tracheal vascular supply to prevent devascularization and ischemic complications. The cricothyroid muscles are detached off the cricoid cartilage and reflected over the cricothyroid joints for added nerve protection.

**Figure 2:**
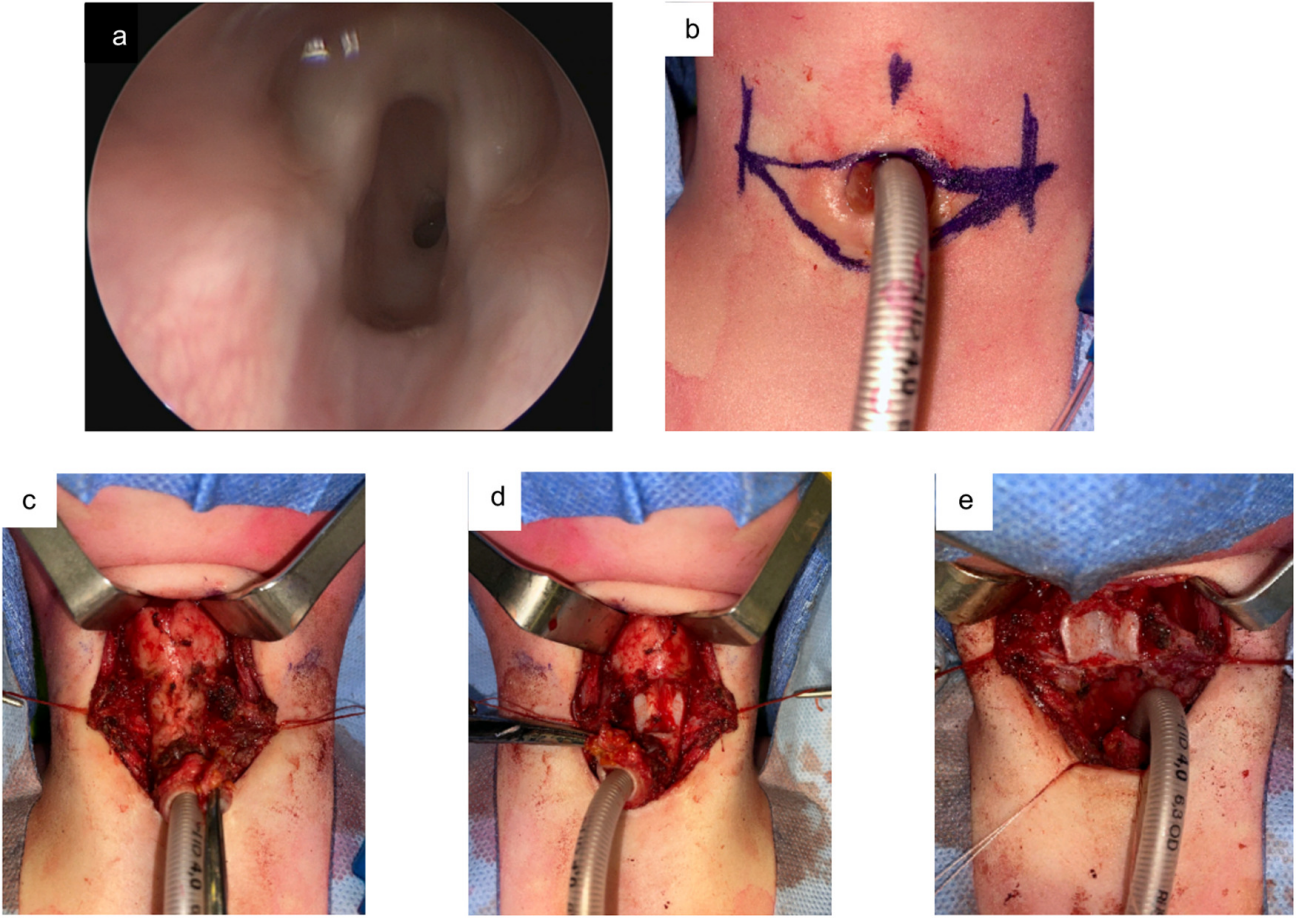
Single-stage PCTR for SGS in a 3-year-old female patient. **(a)** Preoperative endoscopic assessment shows a SGS grade IIIa. **(b)** Skin-resection design around the tracheostoma. **(c)** Exposure of the trachea. **(d)** View after resection of the anterior cricoid arch. **(e)** Status after resection of the stenotic segment.

A partial laryngeal release is performed by incising the thyrohyoid membrane to facilitate superior airway mobilization. Resection of the stenotic segment begins with a superior incision at the inferior margin of the thyroid cartilage, passing laterally anterior to the cricothyroid joint (Figure 2). The posterior subglottic mucosa is incised transversely above the upper limit of the stenotic segment, and the stenosis is dissected in a subperichondrial plane to maximize mucosal preservation. In non-tracheostomized children, the membranous portion of the trachea is dissected free from the anterior esophageal wall over a distance matching the vertical extent of the cricoid plate. A wedge of the anterior tracheal wall is preserved to facilitate subglottic expansion. The inferior limit of resection is established at the caudal edge of the stenotic segment, or—when a single-stage procedure is performed in tracheostomized patients—one ring below the stoma. At this point, the armored endotracheal tube is redirected from the tracheostoma into the distal tracheal segment to secure airway management.

Given the discrepancy in luminal diameter between the proximal subglottic airway and the distal tracheal stump, subglottic widening is necessary in children in order to achieve a well-matched anastomosis without compromising phonatory function. This is accomplished by performing an inferior midline thyrotomy below the anterior commissure to widen the subglottis. The cricoid plate is further expanded posteriorly and laterally using a diamond burr to create a flat surface, which is subsequently covered with the membranous trachea to reduce the risk of restenosis. Additional subglottic expansion is achieved by suturing the lateral subglottic mucosa to the inferior margin of the thyroid cartilage with vicryl sutures.

In cases of PCTR where the vertical incision extends to the petiole of the epiglottis, it is recommended to suture the epiglottis to the tongue base and thyroid cartilage to prevent epiglottic prolapse and ensure airway stability. Additionally, subglottic lateral sutures may be utilized to further enhance stability and maintain patency of the airway.

The thyrotracheal anastomosis begins with the placement of two posterolateral 3.0 vicryl or PDS sutures, which are passed through the posterolateral aspect of the second normal tracheal ring, the posterolateral subglottic mucosa, and the lateral cricoid plate. The posterior anastomosis is then completed using interrupted 4.0 vicryl sutures, or a running PDS 5.0 suture. Fibrin glue (Tisseel ®) is applied to reinforce the suture line and secure the membranous trachea to the cricoid plate. In single-stage procedures, the nasotracheal tube is than advanced into the distal tracheal stump. The anterior anastomosis is established with additional interrupted 4.0 vicryl or PDS sutures between the first/second tracheal ring(s) and the thyroid cartilage. To address the triangular subcommissural defect, a pedicled cartilage wedge, harvested from the first normal tracheal ring and lined with mucosa, is inserted and secured to the thyroid cartilage. A final application of fibrin glue is performed anteriorly to reinforce the closure, and the thyroid lobes are resutured at the midline. A Penrose drain is positioned inferior to the anastomotic site, followed by reapproximation of the strap muscles and closure of the skin in two layers.

**Figure 3:**
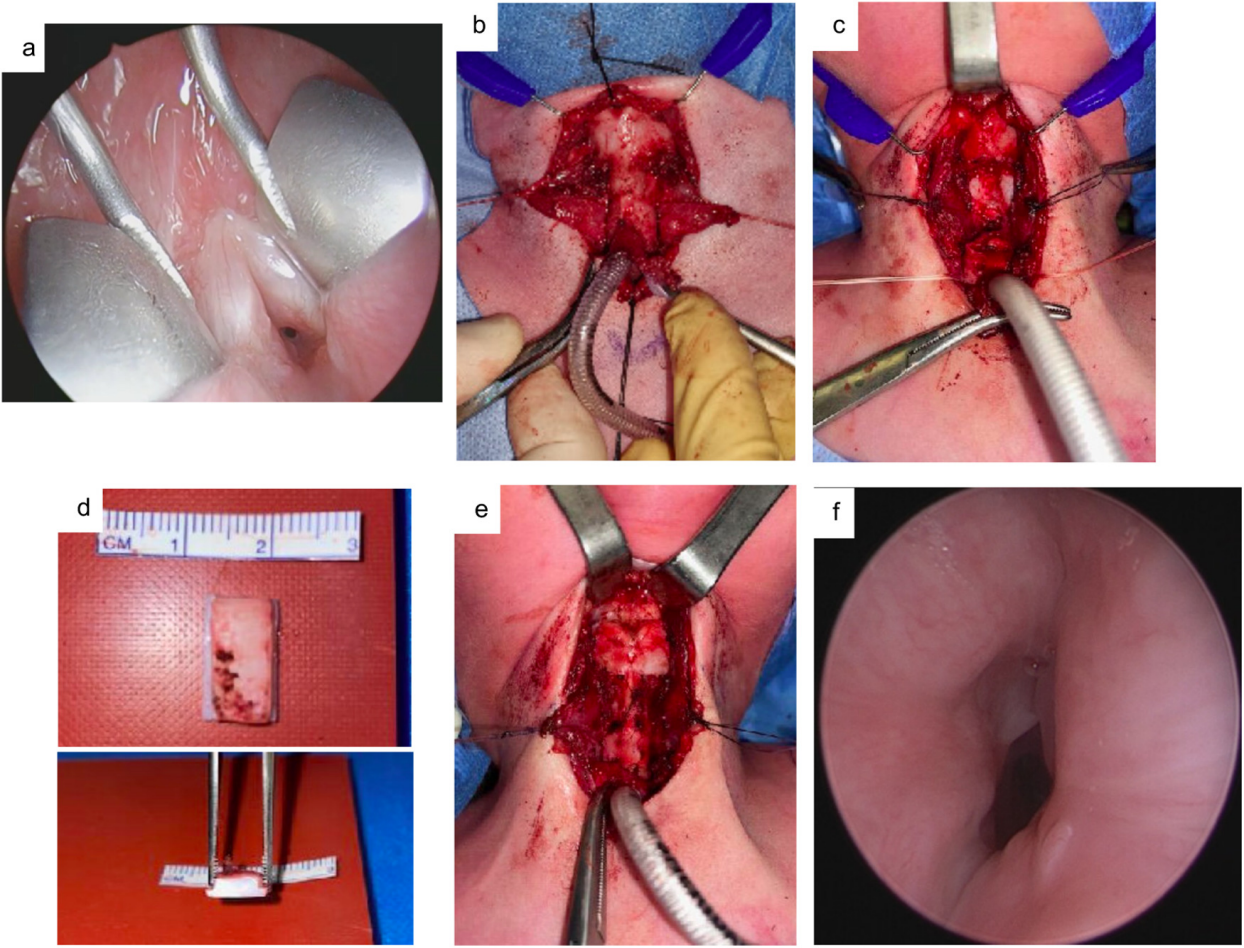
Double-stage extended PCTR for grade IIIb glotto-subglottic stenosis in a 2-year-old male patient. **(a)** Preoperative image. **(b)** Careful dissection of the trachea is performed. **(c)** Resection of the LTS including posterior cricoid split. **(d)** Dimensions of the cartilage graft, harvested from the eighth rib. **(e)** Closure of the thyrotracheal anastomosis. **(e)** View with direct laryngoscopy at four months postoperative. The patient could be decannulated the day after.

In extended PCTR, a double-stage approach is advised (Figure 3). If the initial tracheostomy is located near the subglottis, it is excised, and upon completion of the posterior anastomosis, a new tracheostomy is created three to four rings lower. In extended PCTR, a complete laryngofissure is performed, with division of the airway at the level of the epiglottis. A vertical midline incision is made through the posterior cricoid plate, with complete transection of the interarytenoid muscle. Preservation of phonatory function requires caution to prevent over-widening of the interarytenoid space, which can contribute to a breathy voice. A costal cartilage graft, harvested from the eighth rib, is interposed with its perichondrium facing the tracheal lumen, and is secured using lateral flanges. The graft is stabilized with vicryl sutures to the divided cricoid laminae. A pedicled flap of membranous trachea is harvested through partial resection of one or two additional rings of the tracheal stump, and is sutured to the posterior laryngeal commissure.

Stenting (e.g., LT-Mold ®, Montogomery tube) is often used postoperatively after extended PCTR to support the posterior cartilage graft and prevent restenosis. However, due to the limited availability of suitable pediatric stents, we generally omit stent insertion, without negative consequences on postoperative outcomes. Reconstitution of the anterior commissure while closing the supraglottic portion of the laryngofissure is performed carefully to preserve phonatory function.

### Endoscopic posterior cricoid split and costal cartilage graft insertion

EPCS/RG, first introduced by Inglis et al. in 2002, is a minimally invasive option for selected cases of PGS, SGS, and BVFI (14). It aims to widen the posterior glottis by splitting the posterior cricoid plate and inserting a costal cartilage graft via endoscopy.

The procedure is performed under general anesthesia, typically via a pre-existing tracheostomy. If a tracheostomy is not present, a temporary one is created prior to surgery for airway security. A Lindholm laryngoscope is used to expose the posterior glottis, and an inverted self-retaining retractor (Karl Storz) is applied to lateralize the vocal cords.

A posterior midline cricoidotomy is performed using cold instruments or a CO_2_ laser in continuous high-energy pulse mode, carefully avoiding damage to the interarytenoid musculature and esophageal mucosa. A limited subperiosteal tunnel is developed behind the posterior cricoid plate using a microlaryngeal spatula to accommodate the rib graft flanges. Once the required length and width of the split are measured, the patient is temporarily removed from laryngeal suspension to facilitate costal cartilage harvest.

Rib cartilage is harvested (typically from the seventh rib), preserving the anterior perichondrium. Grooves are carved along both sides with a number 11 blade to form posterior flanges. The groove width is tailored to match the thickness of the cricoid lamina, ensuring optimal fit within the split. A 3.0 silk safety suture is applied through the superior portion of the graft for retrieval in case of accidental dislodgement.

**Figure 4:**
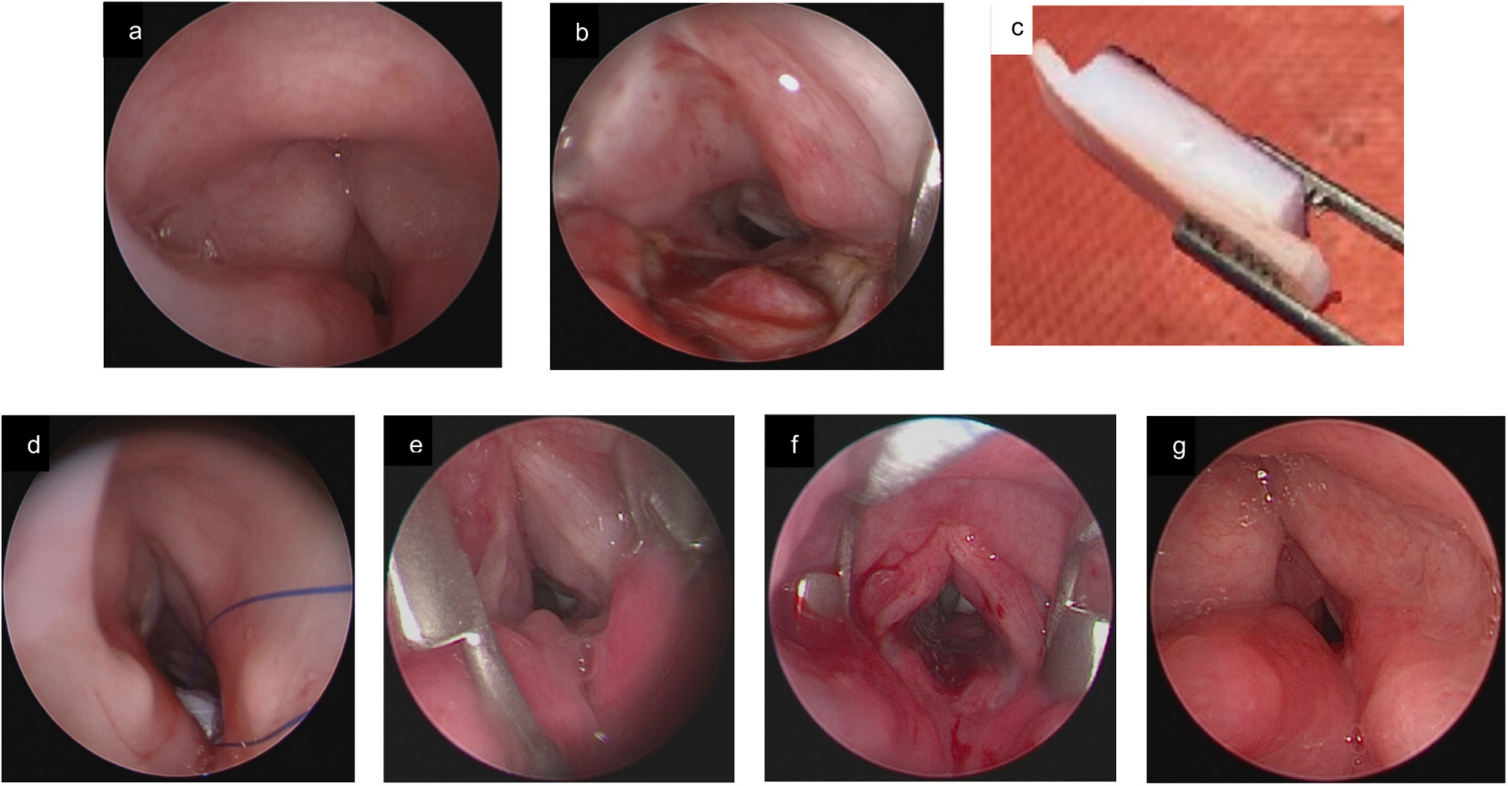
**(a)** preoperative endoscopic image showing a grade III postintubation PGS in a 2-year-old child. **(b)** Endoscopic view after performing a posterior midline cricoidotomy, with visible protrusion of esophageal mucosa. **(c)** Design of the costal cartilage graft. **(d)** Rib placement by using a safety suture. **(e)** Endoscopic image 2 months postoperative. **(f)** Airway status after balloon dilation. **(e)** 3.5 months postoperative. Decannulation could follow the day after.

**Figure 5:**
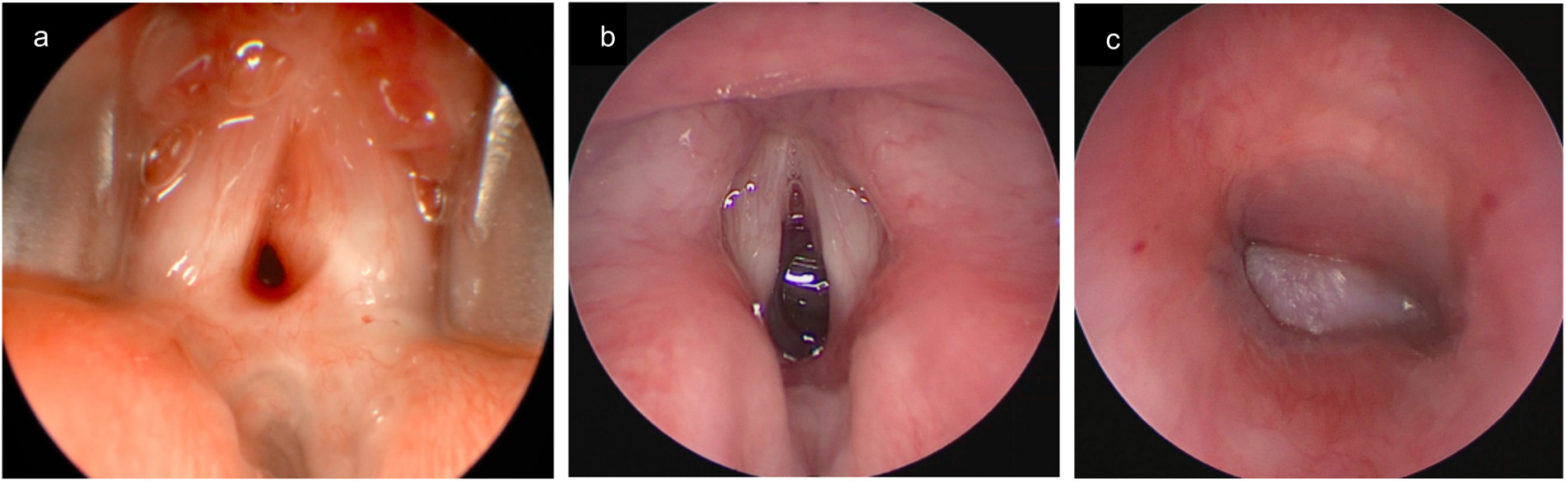
**(a)** preoperative endoscopic image showing a PGS grade IV in a 2-year-old patient. **(b)** Postoperative endoscopic image after six weeks shows a patent airway. **(c)** During the same procedure, a large suprastomal granuloma was noted, which was resected through the tracheostomy.

**Figure 6:**
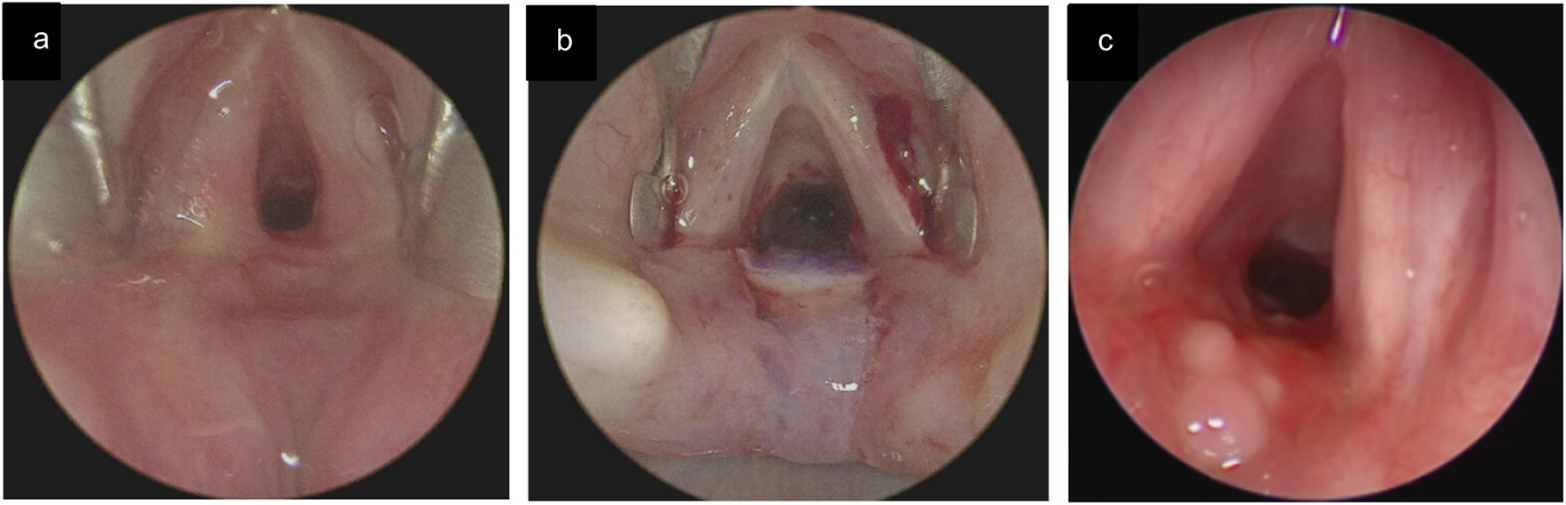
**(a)** endoscopic assessment shows a grade II PGS in an 8-year-old child after stump laryngeal trauma with tracheal rupture and subsequent prolonged endotracheal intubation. **(b)** Confirmation of correct graft position, aligned with the laryngeal lumen. **(c)** Direct laryngoscopy 6 weeks postoperative shows a patent airway with complete epithelialisation of cartilage, and limited interarytenoidal granulation tissue.

The graft is positioned endoscopically with microlaryngeal forceps and wedged firmly into the split cricoid. Proper fit is confirmed endoscopically before the safety suture is removed. No stent is required, as the graft is held in place by the snug friction fit and posterior flanges (13) (Figures 4–6).

## Postoperative management

Postoperative care is critical to the success of airway reconstruction in pediatric patients. Although protocols vary depending on the surgical technique and institutional preferences, core principles remain consistent across procedures.

Patients are typically admitted to the Pediatric Intensive Care Unit (PICU) immediately after surgery for close monitoring. Key early goals include airway stabilization, pain management, prevention of infection, and maintenance of optimal respiratory function.

Daily examination of the neck is necessary to detect signs of subcutaneous emphysema, hematoma, seroma, or wound infection. Imaging may be required if pneumothorax, atelectasis, or pneumonia is suspected.

Antibiotic prophylaxis is initiated perioperatively and tailored according to preoperative culture sensitivities. Antibiotics are continued for a minimum of one week postoperatively. Proton pump inhibitors are routinely administered for at least six weeks to support mucosal healing and prevent acid-related irritation. Nutritional support via nasogastric feeding is initiated as soon as possible after surgery to promote recovery.

In single-stage LTR, nasotracheal intubation is maintained for approximately seven days postoperatively. Extubation is performed under direct laryngobronchoscopic visualization following a 24-hour course of intravenous corticosteroids to minimize airway edema. Follow-up bronchoscopy is typically scheduled at six weeks, with additional assessments based on clinical status. In double-stage LTR, the duration of stenting remains variable and is guided by the extent of reconstruction and airway healing. Intralaryngeal stents (e.g., Montgomery T-tube or LT-Mold ®) are usually maintained for a minimum of six weeks and removed endoscopically. For PCTR, postoperative care focuses on avoiding neck hyperextension to prevent anastomotic dehiscence. Sedation with spontaneous respiration or mild ventilation is maintained for 5–7 days to allow healing. In single-stage PCTR, extubation is attempted after endoscopic assessment under general anesthesia on day seven. Intravenous corticosteroids are started the day before extubation to reduce upper airway edema from prolonged intubation and are continued as necessary. If signs of glottic edema or granulation are present, extubation may be deferred. Post-extubation support may include continuous positive airway pressure (CPAP) to counteract dynamic collapse during vocal cord swelling. After double-stage PCTR, decannulation is deferred until stent removal and airway re-evaluation.

For EPCS/RG, a flexible laryngobronchoscopy is performed seven days postoperatively to assess graft integration and granulation. A direct laryngoscopy follows at around six weeks to evaluate healing and determine readiness for decannulation, which, if feasible, is performed within 24–48 hours.

Routine endoscopic follow-up is recommended in all surgical modalities to detect complications such as restenosis, graft displacement, or granulation tissue formation. Additional endoscopic interventions, if needed, are typically performed under suspension microlaryngoscopy. Dilation of the anastomotic site should be avoided until after the sixth postoperative week.

## Potential complications

Postoperative complications following airway surgery in children vary depending on the technique used, patient-specific factors, and the complexity of the underlying pathology.

In LTR, complications are often associated with suboptimal preoperative assessment, poor surgical technique, or inappropriate patient selection. Respiratory complications may include atelectasis, pneumothorax, aspiration, accidental extubation, or post-extubation stridor. Surgical site infections, hematomas, and seroma formation can also occur, along with wound-related issues at both the cervical and donor sites. Graft-specific complications include prolapse, dehiscence, and tracheocutaneous fistula formation, especially in cases of inadequate fixation or poor healing (15). Restenosis remains a significant concern, particularly in cases involving high-grade SGS or insufficient mucosal coverage.

In PCTR, while overall success rates are high, the procedure carries unique risks. Anastomotic dehiscence is among the most serious complications and may result from excessive tension at the anastomotic site, technical error, local infection, or poor tissue quality (16). Injury to the RLNs, although uncommon, may occur during tracheal dissection. Other reported complications include wound infection, cervical hematoma, and pulmonary complications such as bronchopneumonia and lobar atelectasis. In double-stage PCTR, additional complications may include suprastomal collapse, localized tracheomalacia, and granulation tissue at the tracheostoma site, which can necessitate revision surgery (17).

EPCS/RG-related complications include graft extrusion or migration, particularly in patients who are not tracheostomized at the time of surgery. The risk is mitigated by using posterior flanges and securing the graft firmly within the cricoid split. Other complications include airway granulation, aspiration of dislodged graft material, and, in rare cases, voice alteration. However, since EPCS/RG does not involve direct manipulation of the vocal folds, phonatory function is generally well preserved.

While all surgical techniques carry inherent risks, meticulous technique, vigilant monitoring, and individualized postoperative care can significantly reduce the incidence and severity of complications.

## Surgical outcomes

Surgical outcomes in the management of pediatric LTS are influenced by a complex interplay of disease severity, anatomical site involvement, surgical technique, and perioperative care (17). While decannulation remains the most commonly reported marker of surgical success, functional outcomes—including voice, swallowing, and overall airway stability—are increasingly recognized as equally important metrics, particularly in growing children.

### Laryngotracheal reconstruction

LTR remains a standard approach for moderate-grade SGS, with high decannulation rates reported in several single-center and multicenter series. In a retrospective cohort of 199 children undergoing open airway surgery, Bajaj et al. reported symptomatic improvement in 87.9% of patients, with decannulation rates exceeding 86% across both single-stage and two-stage LTR groups (18). Higher success rates were observed in lower-grade stenosis (18). Similar trends were observed in the study by Elobski et al., which showed high decannulation rates (97.4%) in tracheostomized children, with only one requiring revision LTR (19). A systematic review by Awadh et al., including 108 studies, confirmed an overall LTR success rate of approximately 87% using anterior cartilage grafts (17).

Comparative studies consistently suggest that single-stage LTR is associated with higher decannulation rates than double-stage approaches (20–22). However, this may partly reflect selection bias, as single-stage procedures are typically performed in children with less extensive stenosis and fewer comorbidities. Nevertheless, double-stage procedures remain essential in medically fragile patients, those with extensive stenosis, or where staged healing is necessary.

### Partial cricotracheal resection and extended partial cricotracheal resection

PCTR has emerged as the procedure of choice for severe grade III-IV subglottic stenosis, particularly in patients with limited mucosal integrity or failed previous reconstruction. In their landmark study, Yamamoto et al. evaluated 129 children undergoing PCTR for grade II, III and IV SGSs, and reported an overall decannulation rate of 90% (23). Among these, single-stage procedures yielded a 96% success rate, compared to 81.4% in double-stage cases (23). Glottic involvement was a significant negative predictor of surgical outcome and often necessitated extended resections or combined approaches (23). Similar findings were reported by George et al., who found significantly lower decannulation rates and longer decannulation intervals in children with combined glotto-subglottic stenosis, despite comparable revision rates (24).

Bajaj et al. contributed additional insight with a smaller PCTR cohort (*n* = 13), in which all eight patients undergoing single-stage resection demonstrated both symptomatic and endoscopic improvement (18). In contrast, only three of five patients in the double-stage group achieved decannulation (18). These findings align with those from the Lausanne group and reinforce the role of single-stage PCTR in selected patients without glottic extension or significant comorbidities (23).

Functional outcomes following PCTR have also been evaluated in the literature. Yamamoto et al. reported that over 85% of children experienced no or only mild exertional dyspnea, dysphonia, or dysphagia following PCTR at long-term follow-up (23). However, in a related study, Timman et al. observed that although most patients achieved successful decannulation after laryngotracheal resection, approximately 34% continued to exhibit mild-to-moderate dysphonia despite preserved recurrent laryngeal nerve function (25). The authors hypothesized that this voice alteration might be due to changes in the length of the cricothyroid muscle belly, resulting from surgical modifications to the laryngeal framework. Such alterations could affect the tension and elasticity of the vocal folds, leading to compromised phonatory function (25). These findings underscore the importance of evaluating long-term functional outcomes—particularly voice and swallowing—alongside decannulation success in children undergoing PCTR.

### Endoscopic posterior cricoid split and costal cartilage graft insertion

EPCS/RG offers a less invasive alternative for LTR in selected patients with PGS, BVFI, or selected SGS, particularly in tracheostomized children. Initial results from Inglis et al. reported a 60% decannulation rate, though subsequent studies have shown improved outcomes (14). Dahl et al. reported a decannulation rate of 65.6% in 32 children, which increased to 80.8% when excluding patients with severe comorbidities (11). Gerber et al. published a multi-institutional study of 28 patients, achieving an 89% rate of airway stabilization without need for further surgical intervention (26).

Redmann et al. expanded on this by evaluating outcomes in both primary and revision EPCS/RG (27). Among 21 patients, decannulation was achieved in 85% of primary procedures and 100% of revisions, with no statistically significant difference between the two groups (27). This confirms the feasibility of revision endoscopic grafting even in previously treated airways.

Although the literature reflects variability, outcomes tend to be most favorable in children with isolated PGS and no significant comorbidities (11).

## Conclusion

The surgical management of pediatric LTS continues to evolve, guided by a deepening understanding of airway anatomy, advances in surgical technique, and growing experience with multidisciplinary care models. While no single intervention is universally applicable, current evidence supports a stratified approach, with endoscopic techniques reserved for mild cases, LTR favored in moderate unilevel SGS, and PCTR reserved for severe or recurrent SGS. EPCS/RG provides a promising minimally invasive alternative for LTR in selected cases of PGS.

Essential for success is a rigorous preoperative work-up, including standardized airway assessment and classification, as well as evaluation of comorbidities that may influence healing and function. Just as critical is the postoperative phase, where meticulous care, structured surveillance, and timely intervention for complications can markedly influence outcomes.

Ultimately, the choice of technique must be tailored to the individual child, balancing anatomical complexity, physiological resilience, and institutional expertise. The integration of multidisciplinary airway teams—including otolaryngologists, anesthesiologists, pulmonologists, intensivists, and speech-language specialists—is crucial to achieving optimal outcomes.

As surgical techniques become more refined and evidence-based algorithms are increasingly being adopted, the prospects for children with LTS continue to improve. Future research should focus on long-term functional outcomes, standardized reporting of surgical results, and innovation in minimally invasive and tissue-engineered airway solutions. Through continued collaboration and high-quality clinical research, the goal of safe, effective, and individualized care for every child with airway stenosis becomes increasingly achievable.
